# Supplementary material for: Cellular porosity in dentin exhibits complex network characteristics with spatio-temporal fluctuations
Source: PLoS One. 2025 Jul 16;20(7):e0327030. doi: 10.1371/journal.pone.0327030 (PMC12266439; doi:10.1371/journal.pone.0327030)
Supplement: S1 Fig — a) Graph cleaning pipeline. b) Detailed description of the branch bridges removal conditions (all must be fulfilled). (PDF) [file pone.0327030.s001.pdf]

Graph cleaning algorithm

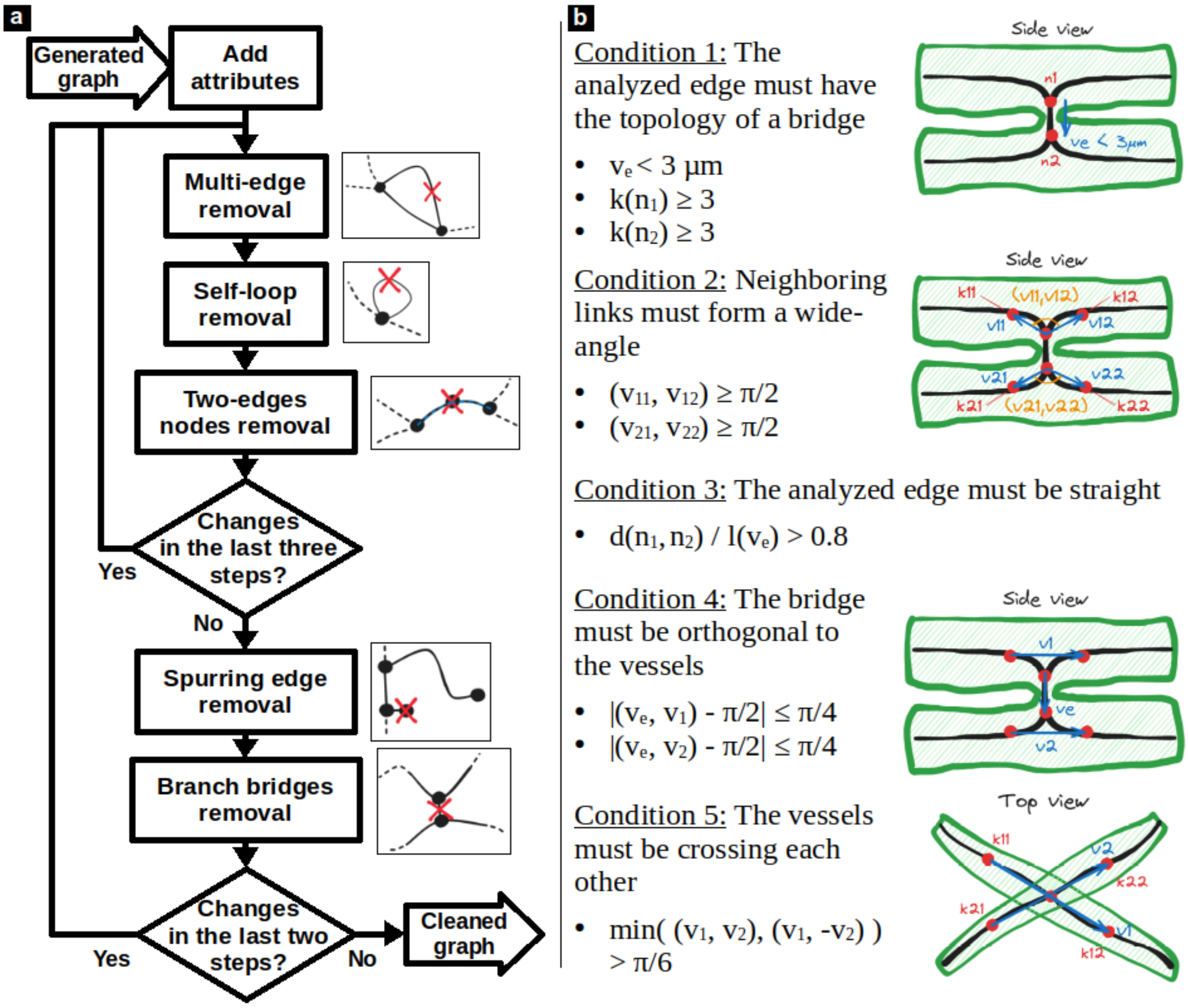

S1 Fig: Graph cleaning algorithm. a) Graph cleaning pipeline. b) Detailed description of the branch bridges removal conditions (all must be fulfilled).
