# Supplementary material for: Cellular porosity in dentin exhibits complex network characteristics with spatio-temporal fluctuations
Source: PLoS One. 2025 Jul 16;20(7):e0327030. doi: 10.1371/journal.pone.0327030 (PMC12266439; doi:10.1371/journal.pone.0327030)
Supplement: S2 Fig — Hatched parts correspond to nodes of degree 1 that are close to the border, indicative of vessels being cut by the borders of the acquisition. (PDF) [file pone.0327030.s002.pdf]

<u>Degree distribution of generated, cleaned and ground-truth graphs</u>

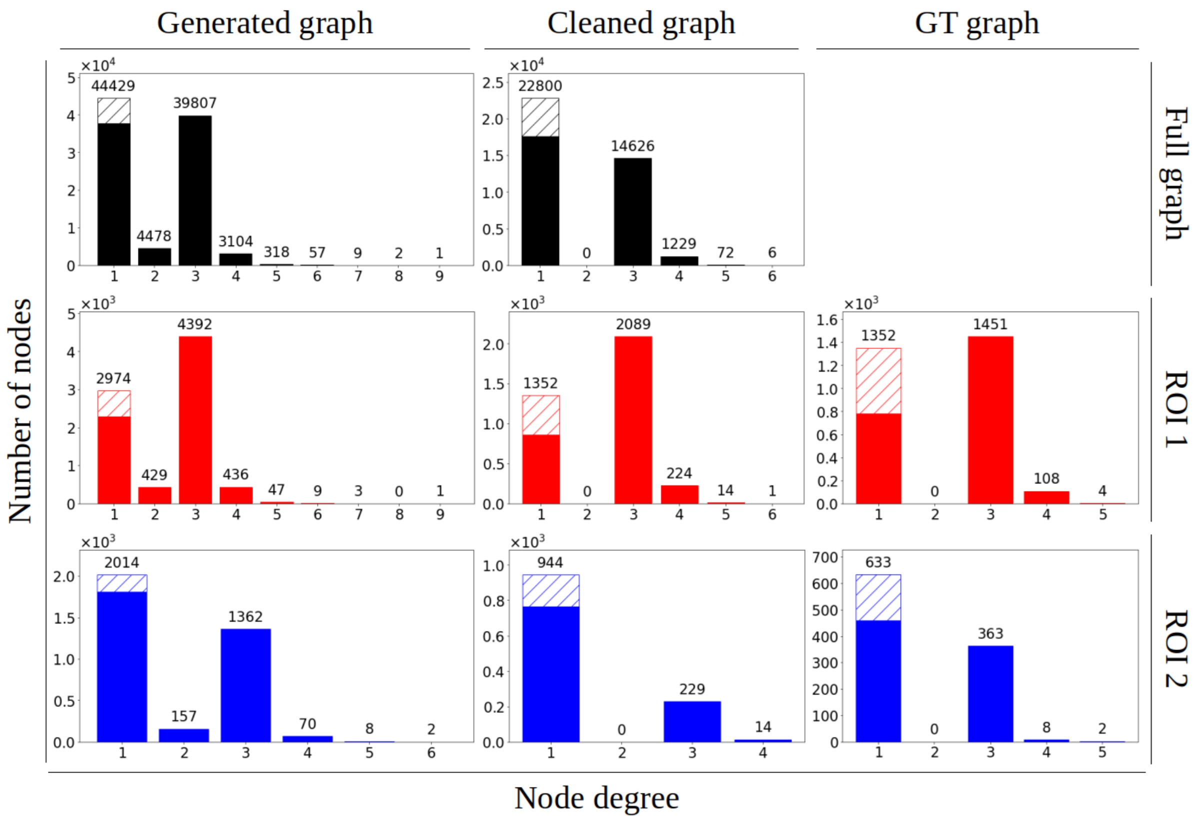

S2 Fig: Degree distribution of the different graphs used in the study. Hatched parts correspond to nodes of degree 1 that are close to the border, indicative of vessels being cut by the borders of the acquisition.
