# Supplementary material for: Cellular porosity in dentin exhibits complex network characteristics with spatio-temporal fluctuations
Source: PLoS One. 2025 Jul 16;20(7):e0327030. doi: 10.1371/journal.pone.0327030 (PMC12266439; doi:10.1371/journal.pone.0327030)
Supplement: S3 Fig — B) Simulation results for metrics (l), D, DI, S, K and FS. The red and blue curves correspond respectively to the ROI 1 and 2. (PDF) [file pone.0327030.s003.pdf]

Full results for the node disconnections error simulations.

A)

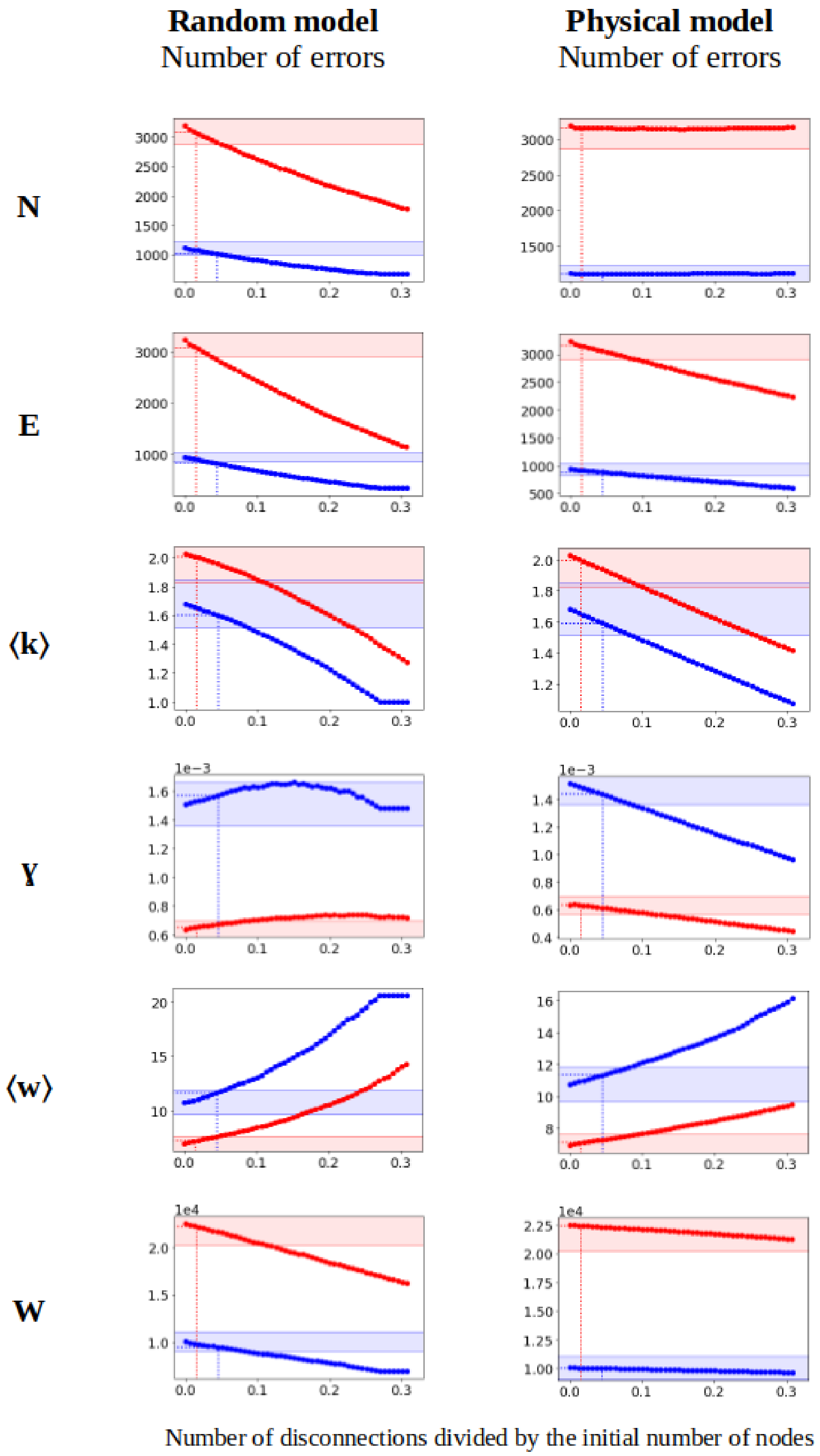

B)

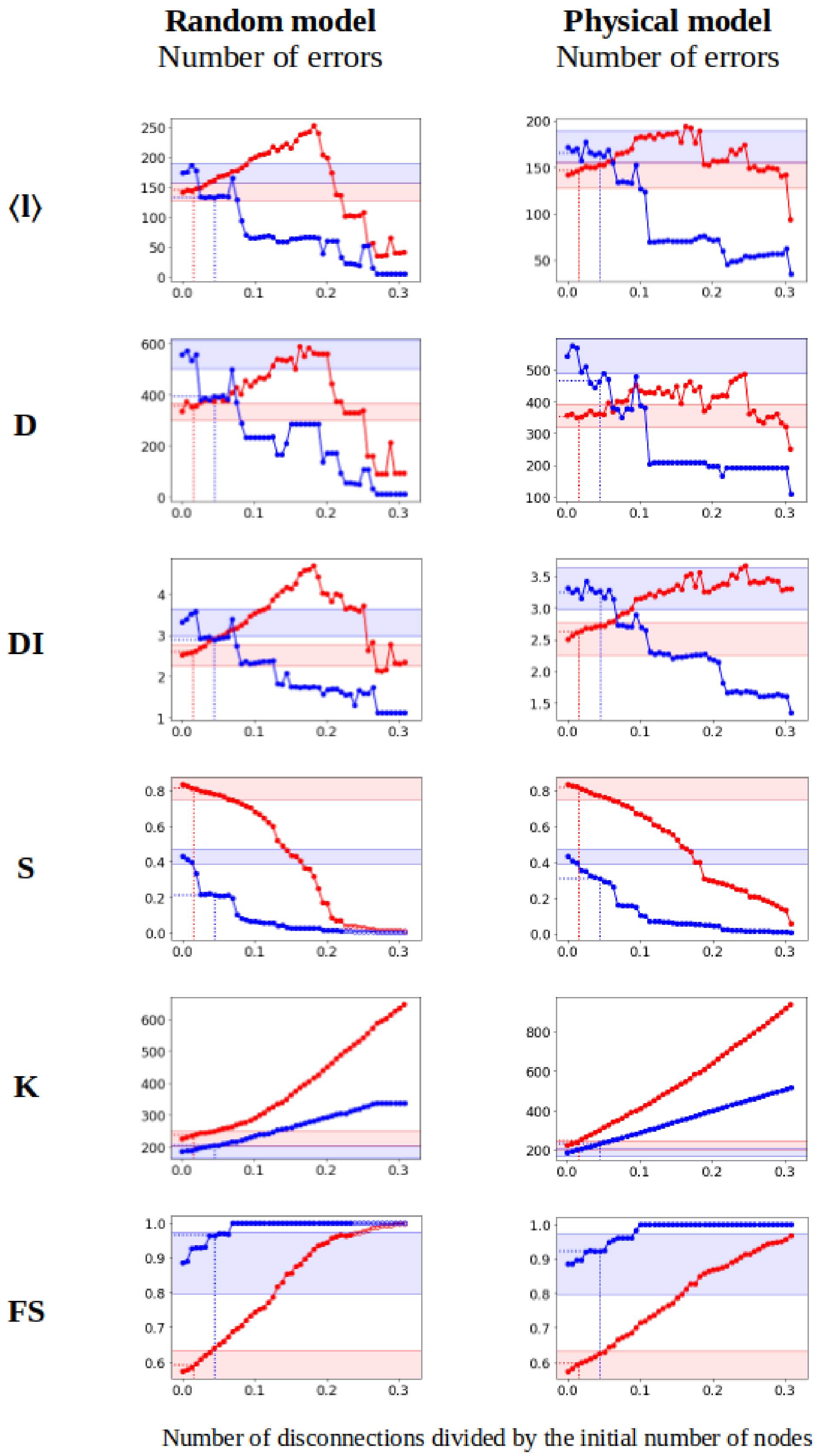

Fig.S3: Node disconnections error simulations: A) results for metrics *N*, *E*, *⟨k⟩*, *γ*, *⟨w⟩* and *W*. B) results for metrics ⟨ l⟩ , *D*, *DI*, *S*, *K* and *FS*. The red and blue curves correspond respectively to the ROI 1 and 2.
