# Supplementary material for: Cellular porosity in dentin exhibits complex network characteristics with spatio-temporal fluctuations
Source: PLoS One. 2025 Jul 16;20(7):e0327030. doi: 10.1371/journal.pone.0327030 (PMC12266439; doi:10.1371/journal.pone.0327030)
Supplement: S4 Fig — The red and blue curves correspond respectively to the ROI 1 and 2. (PDF) [file pone.0327030.s004.pdf]

# Full results for the edge bridges error simulations.

A)

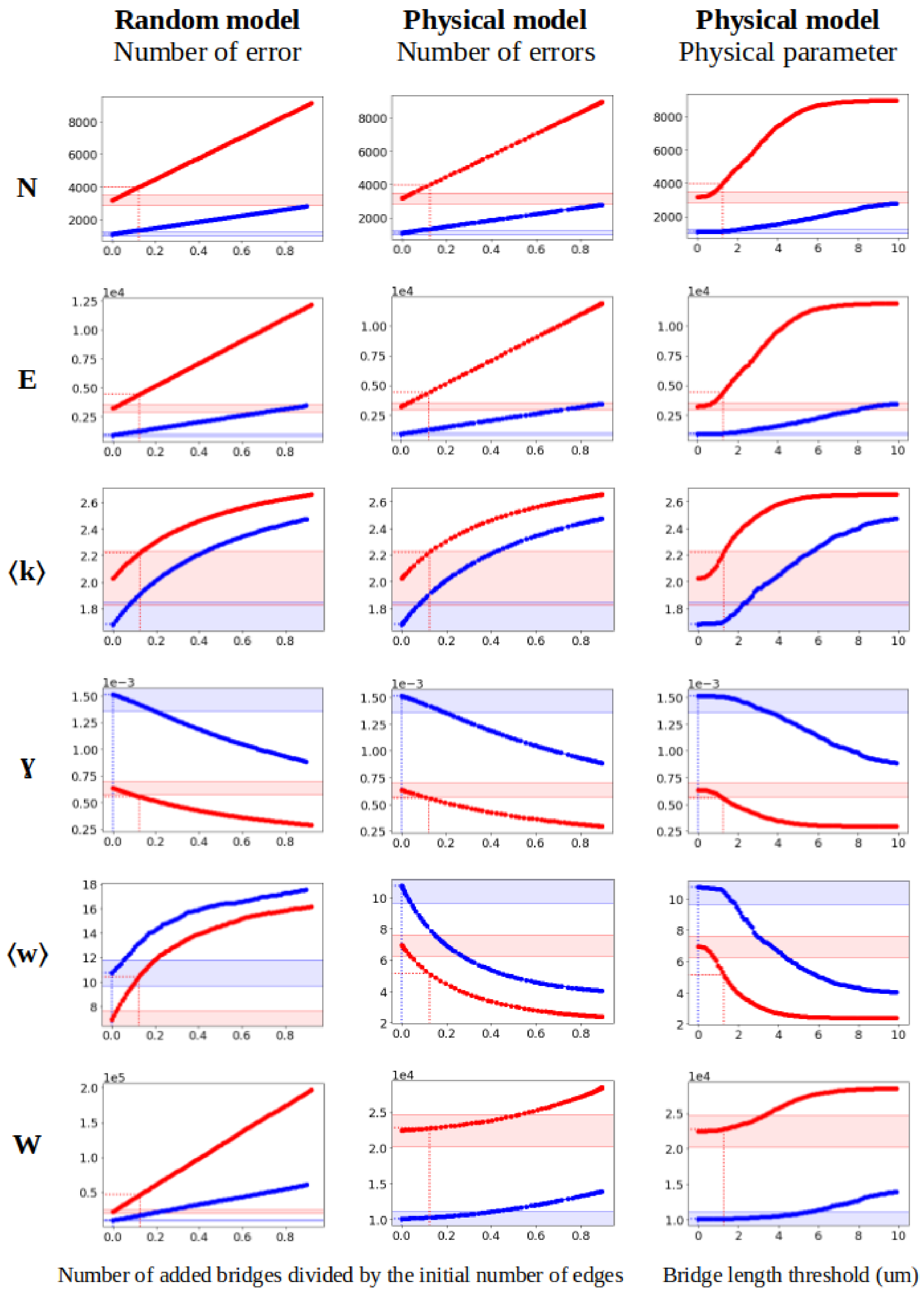

**B)**

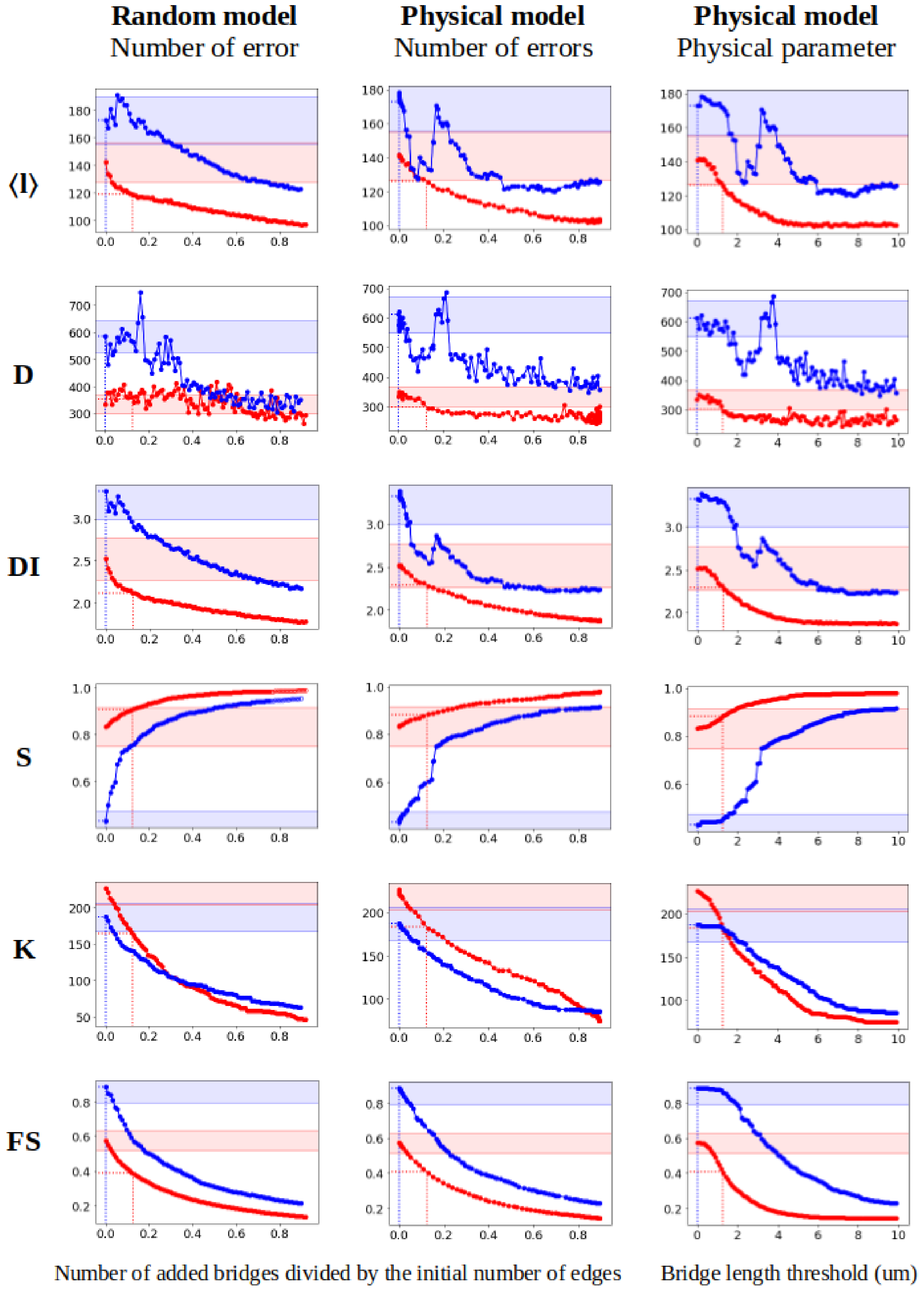


**S4 Fig: Edge bridges error simulations: A) results for metrics *N*, *E*, $\langle k \rangle$, *γ*, $\langle w \rangle$ and *W*. B) results for metrics $\langle l \rangle$, *D*, *DI*, *S*, *K* and *FS*.** The red and blue curves correspond respectively to the ROI 1 and 2.
