# Supplementary material for: Cellular porosity in dentin exhibits complex network characteristics with spatio-temporal fluctuations
Source: PLoS One. 2025 Jul 16;20(7):e0327030. doi: 10.1371/journal.pone.0327030 (PMC12266439; doi:10.1371/journal.pone.0327030)
Supplement: S5 Fig — The red and blue curves correspond respectively to the ROI 1 and 2. (PDF) [file pone.0327030.s005.pdf]

Full results for the missing edges error simulations.

A)

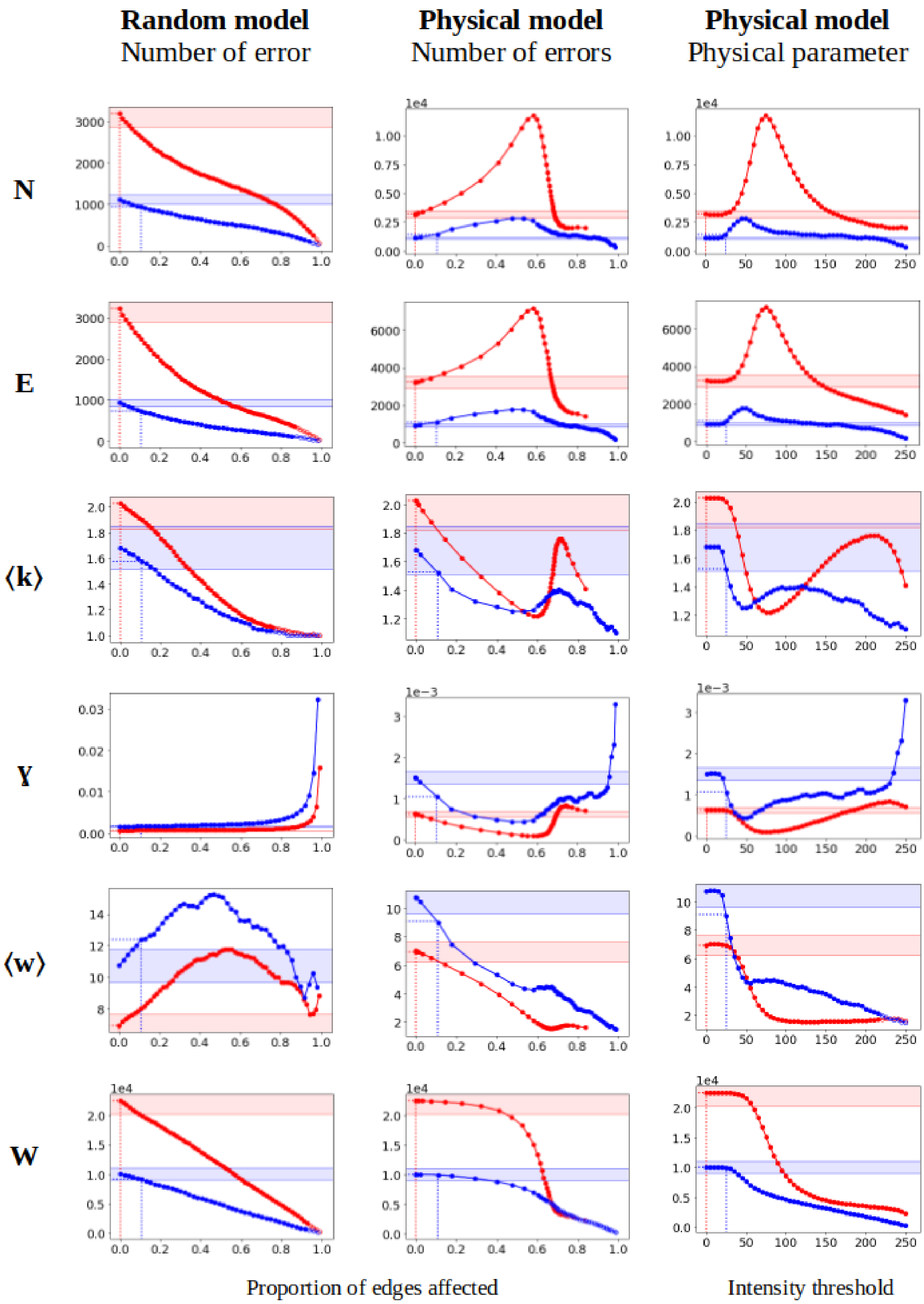

B)

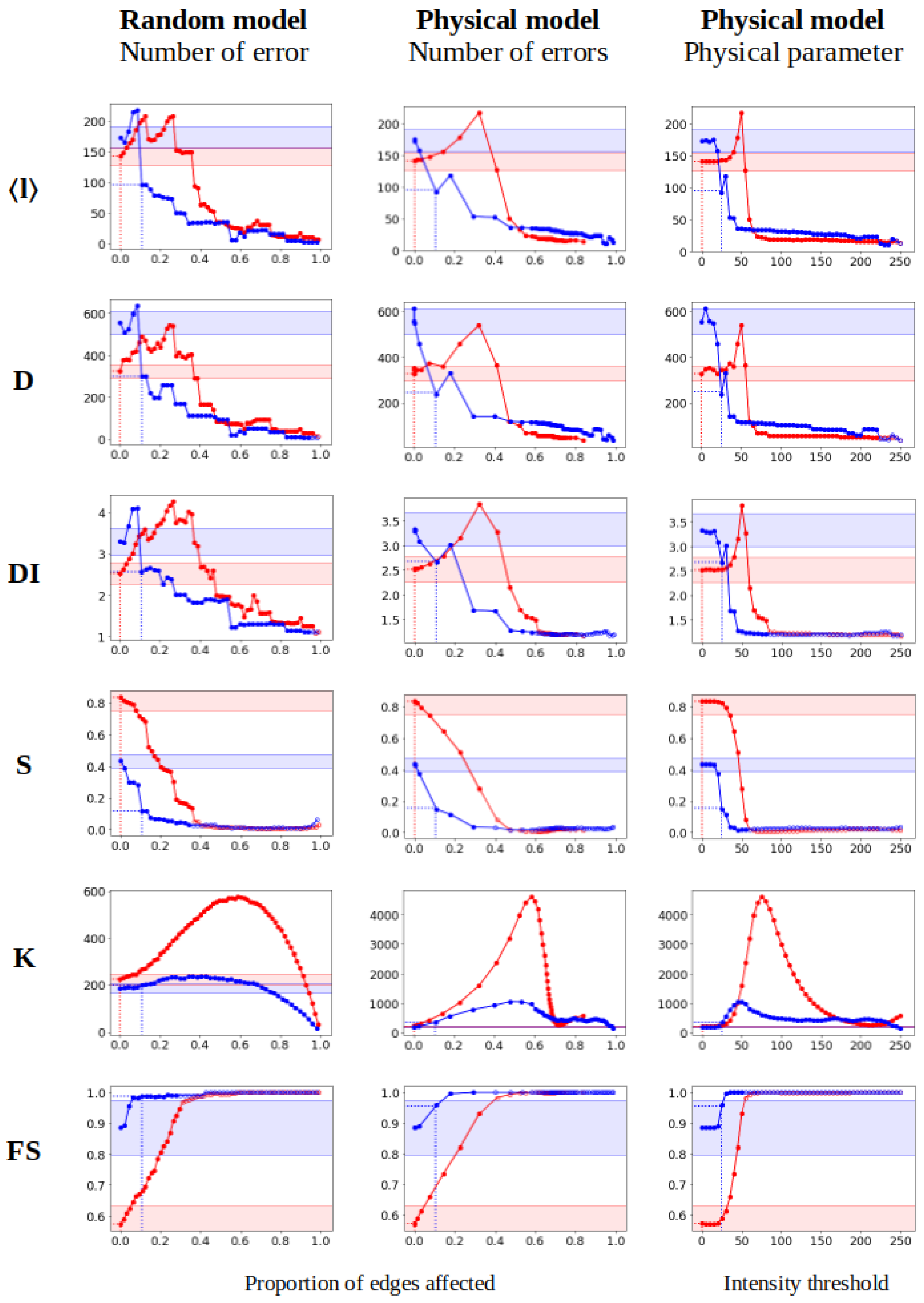

S5 Fig: Missing edges error simulations: A) results for metrics *N*, *E*, ⟨*k*⟩, *γ*, ⟨*w*⟩ and *W*. B) results for metrics ⟨*l*⟩, *D*, *DI*, *S*, *K* and *FS*. The red and blue curves correspond respectively to the ROI 1 and 2.
