# Supplementary material for: Cellular porosity in dentin exhibits complex network characteristics with spatio-temporal fluctuations
Source: PLoS One. 2025 Jul 16;20(7):e0327030. doi: 10.1371/journal.pone.0327030 (PMC12266439; doi:10.1371/journal.pone.0327030)
Supplement: S6 Fig — a) number of edges (E), b) edge density (γ), c) mean shortest path (), d) diameter (D), e) detour index (DI), f) relative size of the largest component (S), g) number of connected components (K). (PDF) [file pone.0327030.s006.pdf]

Metric maps results

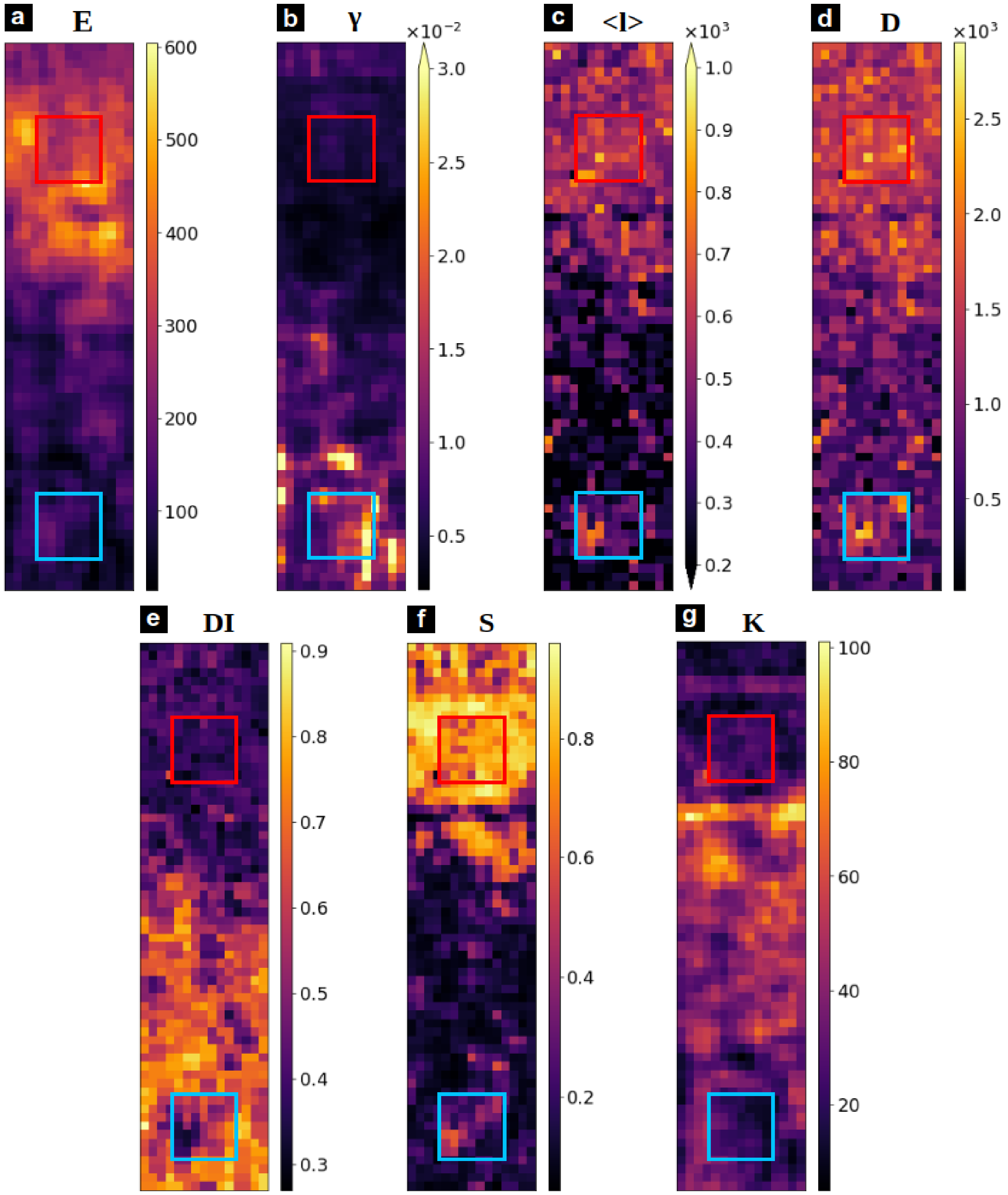

S6 Fig: Spatial maps of network metrics. a) number of edges (***E***), b) edge density (***γ***), c) mean shortest path (***<l>***), d) diameter (***D***), e) detour index (***DI***), f) relative size of the largest component (***S***), g) number of connected components (***K***)
